# Citronellal improves endothelial dysfunction by affecting the stability of the GCH1 protein: CT improves endothelial dysfunction by GCH1 protein

**DOI:** 10.3724/abbs.2024086

**Published:** 2024-06-03

**Authors:** Yaqi Guo, Huadong Que, Bulei Chen, Chunyan Chao, Shanshan Li, Shuang Guo, Yaling Yin, Huanhuan Wang, Moli Zhu, Peng Li

**Affiliations:** 1 SanQuan Medical College Sino-UK Joint Laboratory of Brain Function and Injury and Department of Physiology and Neurobiology Henan International Joint Laboratory of Cardiovascular Remodeling and Drug Intervention School of Basic Medical Sciences College of Pharmacy Xinxiang Medical University Xinxiang 453003 China; 2 Huang Huai University Zhumadian 463000 China; 3 Hubei Key Laboratory of Diabetes and Angiopathy Hubei University of Science and Technology Xianning 437100 China

**Keywords:** citronellal, endothelial dysfunction, Smurf2, degradation

## Abstract

Endothelial dysfunction (ED) serves as the pathological basis for various cardiovascular diseases. Guanosine triphosphate cyclopyrrolone 1 (GCH1) emerges as a pivotal protein in sustaining nitric oxide (NO) production within endothelial cells, yet it undergoes degradation under oxidative stress, contributing to endothelial cell dysfunction. Citronellal (CT), a monoterpenoid, has been shown to ameliorate endothelial dysfunction induced by in atherosclerosis rats. However, whether CT can inhibit the degradation of GCH1 protein is not clear. It has been reported that ubiquitination may play a crucial role in regulating GCH1 protein levels and activities. However, the specific E3 ligase for GCH1 and the molecular mechanism of GCH1 ubiquitination remain unclear. Using data-base exploration analysis, we find that the levels of the E3 ligase Smad-ubiquitination regulatory factor 2 (Smurf2) negatively correlate with those of GCH1 in vascular tissues and HUVECs. We observe that Smurf2 interacts with GCH1 and promotes its degradation via the proteasome pathway. Interestingly, ectopic Smurf2 expression not only decreases GCH1 levels but also reduces cell proliferation and reactive oxygen species (ROS) levels, mostly because of increased GCH1 accumulation. Furthermore, we identify BH _4_/eNOS as downstream of GCH1. Taken together, our results indicate that CT can obviously improve vascular endothelial injury in Type 1 diabetes mellitus (T1DM) rats and reverse the expressions of GCH1 and Smurf2 proteins in aorta of T1DM rats. Smurf2 promotes ubiquitination and degradation of GCH1 through proteasome pathway in HUVECs. We conclude that the Smurf2-GCH1 interaction might represent a potential target for improving endothelial injury.

## Introduction

Vascular endothelial cells play a pivotal role in responding to various stimuli and maintaining vascular homeostasis. Endothelial dysfunction (ED) is characterized by decreased nitric oxide (NO) bioavailability, overproduction of reactive oxygen species (ROS), and inflammation, which are underlying abnormalities in hypertension, coronary artery disease and diabetes [ 1– 3] . Guanosine triphosphate cyclohydrolase 1 (GCH1), a key enzyme catalyzing the production of tetrahydrobiopterin (BH _4_), is involved in the synthesis of numerous hormones and neurotransmitters, playing a vital role in various pathophysiological processes in the body [ 4, 5] . For instance, GCH1 inhibition reduces microglial inflammation [6], and it participates in endothelial dysfunction in atherosclerosis [7]. Several studies have demonstrated that upregulation of GCH1 improves injuries in different types of endothelial cells (ECs), such as brain microvascular [8], palmitic acid-induced islet [9] and high glucose (HG)-induced aortic EC injury [ 5, 10] . Diabetes, recognized as a global public health problem, is an independent risk factor for metabolic and cardiovascular diseases [ 11– 13] . In particular, impaired endothelial cells in type 1 diabetes mellitus (T1DM) contribute to the disruption of vascular homeostasis, leading to ED [ 1, 14, 15] . Decreased NO production or increased oxidative stress in endothelial cells leads to reduced NO bioavailability [ 16, 17] . Alterations in endothelial nitric oxide synthase (eNOS) often result in impaired eNOS formation [18]. ED represents the initial stage in the progression of diabetes-associated vascular complications, correlated with elevated oxidative stress and inflammation [ 19, 20] . Enhancement of GCH1-mediated eNOS recirculation attenuates HD-induced ED; however, the exact mechanism is unclear.

Smad-ubiquitination regulatory factor 2 (Smurf2), a member of the HECT E3 ubiquitin ligase family, plays a regulatory role in ubiquitination-mediated protein degradation [21]. It exerts negative functions in transforming growth factor-β (TGF-β) and bone morphogenetic proteins (BMP) signaling pathways [22]. Smurf2-deficient mice are prone to various cancers, suggesting its potential role as a tumor suppressor due to altered histone modification and chromatin compaction [23]. Recent studies have expanded the range of Smurf2 substrates, revealing its involvement in controlling cell cycle, proliferation, differentiation, metastasis, and senescence [ 24– 26] . In the cardiovascular system, Smurf2 is implicated in the regulation of the transcription factor Yin Yang 1 (YY1), which prevents myocardial differentiation and maintains the proliferation ability of cardiac precursor cells [27]. Serving as the E3 ubiquitination ligase of YY1, Smurf2 modulates the protein stability and transcriptional activity of YY1. Additionally, YY1 and Smad7 can interact and synergistically inhibit TGF-β signaling in the nucleus [28]. Numerous studies have indicated the inseparable connection between oxidative stress damage and ubiquitination [29]. For instance, Smurf2 has been implicated in oxidative stress-induced apoptosis in HUVECs, mitigating the generation of ROS induced by H _2_O _2_. This effect is associated with Smurf2, promoting the ubiquitination of PARP1 and mediating its degradation [30]. Despite these insights, limited information is available on the association between Smurf2 and cardiovascular diseases. Whether GCH1 can serve as a novel substrate for Smurf2, improving oxidative stress-induced endothelial cell damage, remains unclear.

Essential oils are recognized for their safety, high efficacy, and low likelihood of resistance [ 31– 33] . Citronellal (CT), an acyclic monoterpene aldehyde derived from secondary metabolism, can be isolated from the oils of bergais, citronella, and wintergrass, displaying potent antimicrobial effects [ 34– 36] . As studies have reported, CT inhibits *Candida albicans* growth by impacting bacterial membrane integrity and disrupting the bacterial cell cycle [37]. More recently, accumulating evidence suggests that CT essential oils can inhibit ROS levels, thereby mitigating oxidative stress and ameliorating vascular endothelial dysfunction [38]. However, it is unclear whether CT can improve vascular endothelial dysfunction by intervening with the expression of GCH1 protein.

In this study, we found that Smurf2 plays an important role in the ubiquitination and proteasomal degradation of GCH1. GCH1 serves as a novel action substrate for Smurf2 to negatively regulate apoptosis in human umbilical vein endothelial cells (HUVECs). It was also revealed that Smurf2 regulates the stability of GCH1 as well as mediates the degradation of GCH1, which may be related to the ubiquitination proteasome system.

## Materials and Methods

### Cell culture and treatment

HUVECs (ATCC, Manassas, USA) were maintained in endothelial cell medium (ECM; ScienCell, San Diego, USA) supplemented with 10% FBS (Gibco, Carlsbad, USA), penicillin (100 IU/mL), and streptomycin (100 μg/mL; Sigma, St Louis, USA) under the standard culture condition (at 37°C in a 5% CO _2_ humidified atmosphere). HUVECs in the logarithmic growth phase were incubated with 200 μM H _2_O _2_ for 48 h to induce vascular endothelial cell damage. The cells were divided into three groups ( *n*=3): (1) Control group; (2) H _2_O _2_ group (200 μM); and (3) CT (320 μM; Yuke, Beijing, China)+H _2_O _2_ group (200 μM).

### Animals and treatments

Male Sprague-Dawley rats (SD, 6‒8 weeks old, 180‒250 g) were procured from Henan Provincial Laboratory Animal Center (Zhengzhou, China). Animal experiment procedures strictly adhered to the National Institutes of Health’s Guidelines for the Care and Use of Laboratory Animals and were approved by the Animal Protection and Use Committee of Xinxiang Medical College. After one week of adaptive feeding, SD rats were intraperitoneally injected with 60 mg/kg streptozotocin (STZ; Sigma) dissolved in 0.1 M citric acid buffer (pH=4.5) to establish a T1DM model. Fasting blood glucose levels exceeding 16.7 mM after 72 h confirmed successful establishment of the model. T1DM rats were randomly divided into 4 groups: T1DM, T1DM+CT (150 mg/kg/day), T1DM+Lovastatin (4 mg/kg/day; Wansheng, Beijing, China), and T1DM+CT (150 mg/kg/day)+Lovastatin (4 mg/kg/day) [ 39, 40] . Normal rats were divided into Control and Control+CT (150 mg/kg/day) groups. Each group comprised 6 rats, receiving daily gavage for 6 weeks.

### Measurement of fasting blood glucose and blood lipids

Fasting blood glucose (FBG) was measured in blood samples collected from the tail vein of all animals after 12 h of fasting prior to execution. The total cholesterol (TC) and triglyceride (TG) levels in rat serum were determined using commercial biochemical kits purchased from Jiancheng Biological Company (Nanjing, China). Sample absorbance values were determined with a Multiskan™ FC microplate photometer (Thermo Fisher Scientific, Waltham, USA). The TC and TG levels of each serum were calculated according to the formula: (A _sample_‒A _blank_)/(A _standard_‒A _blank_)×C _standard_.

### Endothelium-dependent relaxation (EDR) assay

Rat thoracic aortas were isolated post-anesthesia, cut into 3–4 mm rings, and placed in an organ chamber (95% O _2_, 5% CO _2_, 37°C) filled with Krebs buffer (118.3 mM NaCl, 4.7 mM KCl, 0.6 mM MgSO _4_, 1.2 mM KH _2_PO _4_, 2.5 mM CaCl _2_, 25.0 mM NaHCO _3_, 0.026 mM EDTA, and 11.0 mM glucose, pH 7.4). After a 10-min equilibration, 2.0 g of tension was applied for 90 min, followed by pre-constriction with phenylephrine (1 μM; Sigma). Acetylcholine (Ach; Sigma) or sodium nitroprusside (SNP; Sigma) at varying concentrations induced EDR at the plateau of contraction. Relaxation capacity, expressed as a proportion of maximal Ach-induced relaxation, was quantified.

### HE staining

Thoracic aortas were fixed in 4% paraformaldehyde for 12 h, embedded in paraffin, and then were cut into 7-μm-thick slices. After dewaxing and rehydration, the slices were covered with 1% acid ethanol (1% HCl in 70% ethanol) and then stained with hematoxylin solution (Solarbio, Beijing, China) for 5 min. Subsequently, the slices were stained with eosin solution (Solarbio) for 3 min, dehydrated with gradient alcohol and cleared in xylene. All the sections were photographed and analyzed using an Olympus BX53 fluorescence microscope (Olympus, Tokyo, Japan).

### Masson staining

Thoracic aortas were fixed in 4% paraformaldehyde for 12 h, embedded in paraffin, and sectioned into 7-μm slices. After dewaxing and rehydration, the staining process was carried out according to the instructions of the modified Masson stain kit (Solarbio) as follows: slices were incubated in Bouin’s solutionat 37°C for 2 h. Lazurite blue was applied for 2‒3 min, with subsequent washing and gentle drying. The nuclei were stained with Regaud or Weigert’s hematoxylin for 5‒10 min. The cells were differentiated with 1% hydrochloric acid alcohol for 5‒15 sec, extensively washed, and stained for 10 min with spring red fuchsin. Sections were then differentiated with 1% phosphomolybdic acid solution for 10 min, followed by dyeing with aniline blue or 1% light green aqueous solution for 1‒2 min. After wash with 1% weak acid s solution for 1‒2 min, dehydration occurred with 95% and absolute ethanol. Xylene was used for transparencing, followed by sealing with neutral gum. Sections were dried in the fume hood and examined by microscope.

### Transmission electron microscopy (TEM)

The vascular tissues of rats were rinsed with pre-chilled physiological saline at 4°C, cut into tissue blocks smaller than 3 mm ^3^, and then fixed in glutaraldehyde. After dehydration, infiltration, and embedding in gradient alcohols, samples were prepared into 70-nm-thick electron microscopy sections. These sections were examined using a Philips CM-120 transmission electron microscope (Philips, Eindhoven, Netherlands). The arrangement of endothelial cells and the integrity of nuclear membranes were observed.

### Immunohistochemistry analysis

Sections were dewaxed, subjected to antigen retrieval in 1 mM EDTA (pH=8.0) at 95°C for 30 min, and treated with 3% H _2_O _2_ for 15 min. After overnight incubation with anti-GCH1 and Smurf2 antibodies (1:500; CST, Beverly, USA) at 4°C, sections were washed with 0.1 M PBS 3 times (5 min each), followed by incubation with HRP-conjugated goat anti-rabbit IgG (H+L) secondary antibody (1:500; Servicebio, Wuhan, China) for 1 h. Subsequently, sections were washed 3 times (5 min each), incubated with 3,3′-diaminobenzidine (DAB) chromogen for 5 min at room temperature, and imaged using a light microscope.

### Western blot analysis

Total protein extraction was performed using Triton X-100 lysis buffer (1 mM EDTA, 40 mM Tris-HCl, pH 8.0, 100 mM NaCl, 0.5% Nonidet P-40, and 1% Triton X-100). The protein content was quantified using the BCA protein assay kit (Pierce, Rockford, USA). Equal amounts of samples were electrophoresed on 12.5% SDS-PAGE gel and then transferred to immuno-blot polyvinylidene difluoride membranes (Millipore, Billerica, USA). Membranes were blocked in 5% fat-free milk in Tris-buffered saline with 0.1% Tween 20, followed by incubation with primary antibodies overnight at4°C, after which peroxidase-labeled anti-rabbit or anti-mouse IgG secondary antibodies (Servicebio) were applied, respectively. The signals were detected with an enhanced chemiluminescent solution (Millipore). Protein bands were visualized using Amersham Biosciences Imager 600 (GE Healthcare, Bethesda, USA). The primary antibodies against the following proteins were used: GCH1 (1:1000; Santa Cruz Biotech, Santa Cruz, USA), Smurf2 (1:1000; CST) and GAPDH (1:10,000; Abways, Shanghai, China).

### Co-immunoprecipitation assay

The cells were lysed in Triton X-100 buffer, followed by incubation with appropriate primary antibodies overnight at 4°C, treated with proteasome inhibitor MG132 (Univ, Shanghai, China), and then incubated with Protein G Plus/Protein A-agarose beads (Abxin, Shanghai, China) for 2 h at 4°C. The beads were then washed three times with lysis buffer and boiled in 5× sample loading buffer for 10 min. Cell lysates and immunoprecipitates were subjected to western blot analysis.

### Immunofluorescence staining

The cells grown on coverslips were cultured in medium. The cells were washed twice with PBS, fixed in 4% paraformaldehyde for 10 min, permeabilized with 0.1% Triton X-100 for 5 min at room temperature, blocked in 1% BSA for 30 min at room temperature, and incubated with anti-GCH1 and Smurf2 antibodies (CST) overnight at 4°C. Then, the corresponding fluorescent secondary antibody (1:200; Servicebio) was added and incubated for 30 min at room temperature. After two times wash with PBS, the coverslips were mounted with PBS, and the results were ingested into a 3D digital pathology slide scanner.

### TUNEL staining

Aortic paraffin sections were deparaffinized and treated with 20 μg/mL proteinase K at 37°C for 25 min. Sections were then incubated with 50 μL of TUNEL assay solution (Beyotime, Shanghai, China) for 1 h at 37°C in the dark. After three times PBS washing, cell nuclei were stained with 50 μL of DAPI for 3 min. Fluorescence quencher was added to seal the sections. For HUVECs, methanol was added for 10 min after discarding the medium. After being washed with PBS, cells were treated with 0.3% Triton X-100 for 5 min, incubated with 50 μL of TUNEL working solution at 37°C for 60 min, and stained with 50 μL DAPI for 3 min. Cells were lifted, inverted onto slides with anti-fluorescence quencher, and examined under a fluorescence microscope.

### Statistical analysis

Data are presented as the mean±SEM. Statistical analyses were performed using GraphPad Prism 7.0. One-way ANOVA followed by Tukey *post hoc* tests or Bonferroni *post hoc* analyses were used for multiple comparisons. *P*<0.05 was considered statistically significant.

## Results

### CT reduces FBG and lipid levels in T1DM rats, improving endothelium-dependent vasodilation

**Figure 1:**
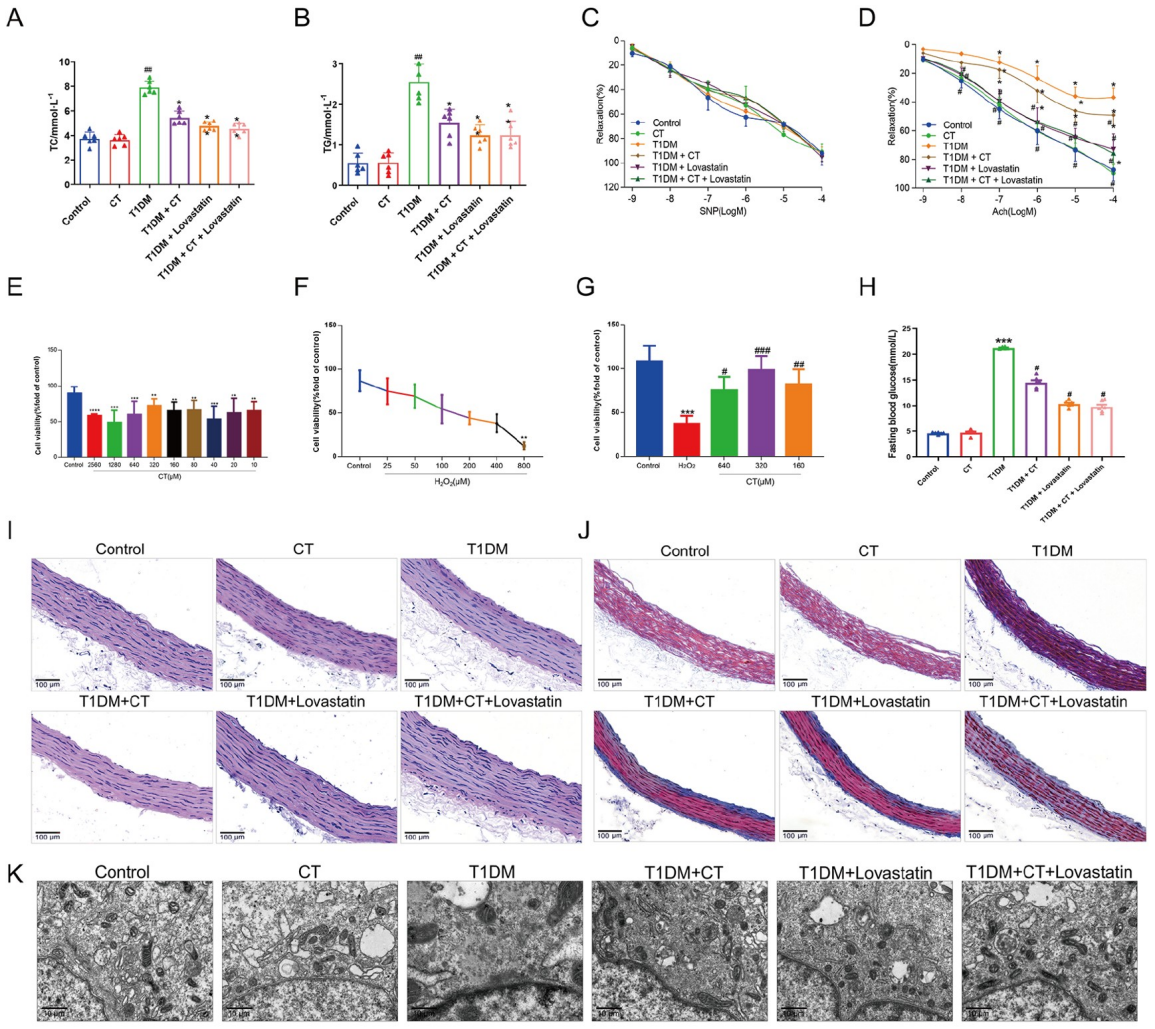
Figure 1 Protective effect of CT on H _2_O _2_-induced endothelial cell injury and the aorta in T1DM rats (A,B) Effect of CT on blood lipid levels in T1DM rats ( n=6). (C,D) Effect of CT on endothelium-dependent relaxation induced by acetylcholine (Ach) or sodium nitroprusside (SNP) in organ lumen ( n=6). (E) Effect of CT on cell proliferation of HUVECs ( n=3). (F) Effect of H 2O 2 on cell viability of HUVECs ( n=3). (G) Survival rate after treatment of H 2O 2-induced endothelial cell injury with different concentrations of CT ( n=3). (H) The effect of CT on FBG level of rats in each group ( n=6). (I) H&E staining of carotid arteries ( n=6). Scale bar: 100 μm. (J) Masson staining of carotid arteries ( n=6). Scale bar: 100 μm. (K) The ultrastructural changes of endothelial injury induced by CT in T1DM rats were observed by TEM ( n=6). Scale bar: 10 μm. Data were analyzed by one-way ANOVA by Tukey post hoc tests or Bonferroni post hoc tests. All data are expressed as the mean ±SEM. * P<0.05 vs Control group, # P<0.05 vs T1DM group or H 2O 2 group.

Endothelial-dependent vasodilator dysfunction is a prominent feature of ED in diabetes, characterized by the impact of hyperglycemia and abnormal lipid metabolism on the vascular endothelium, leading to impaired vascular dilation [41]. Therefore, we measured FBG, TC, and TG levels in rats in each group after modeling and drug administration ( Figure 1A,B,H, *P*<0.05). The results showed that the levels of FBG, TC, and TG in the T1DM group were significantly higher than those in the control group. However, lovastatin, CT, and the combination of lovastatin and CT interventions led to varying reductions in blood glucose and lipid levels in T1DM rats. EDR assessment revealed significant inhibition in T1DM rats compared to the control group, while CT treatment improved EDR ( Figure 1C, *P*<0.05). The impact of CT on SNP-induced vascular relaxation was not significantly different between groups ( Figure 1D). In conclusion, CT’s protective effect on the vascular endothelium is associated with the reduction of blood glucose and lipid levels, ultimately improving EDR.

### CT ameliorates H _2_O _2_-induced injury to HUVECs and endothelial damage in thoracic aorta of T1DM rats

As shown in Figure 1E compared with those in the control group, the endothelial cells in the 10‒640 μM treatment group exhibited no obvious cytotoxicity after 24 h of treatment. CT significantly promoted endothelial cell proliferation in the concentration range of 80‒320 μM, with the strongest promotion observed at 320 μM. CT inhibited endothelial cell proliferation at a concentration of 640 μM. Therefore, 320 μΜ was chosen as the optimal concentration for the study. Figure 1F shows that the treatment of 200 μM H _2_O _2_ for 24 h reduced the survival rate of HUVECs to approximately 50%, indicating a moderate reduction with stable reproducibility. H _2_O _2_ (200 μM) was selected as the model concentration for inducing oxidative damage in HUVECs. To evaluate CT’s protective effect on H _2_O _2_-induced injury, HUVECs were exposed to H _2_O _2_ (200 μM) and CT (640, 320, or 160 μM) for 24 h. As shown in Figure 1G, cell viability was significantly increased in the CT group (320 μM) compared with H _2_O _2_ alone. In addition, in order to explore the effect of CT on ED in T1DM rats, we injected STZ intraperitoneally into SD rats to establish T1DM model. HE staining and TEM revealed disordered carotid endothelial cell arrangement and swollen cell membranes in T1DM rats compared with the control group ( Figure 1I,K). High-concentration CT treatment noticeably alleviated carotid artery injury ( *P*<0.05). Furthermore, Masson staining for collagen deposition ( Figure 1J) exhibited a significant increase in collagen amount in the aorta in the T1DM group compared with the control group. Administration of CT reduced collagen fiber deposition in the carotid artery ( *P*<0.05).

### CT negatively regulates the expression of Smurf2/GCH1 protein

**Figure 2:**
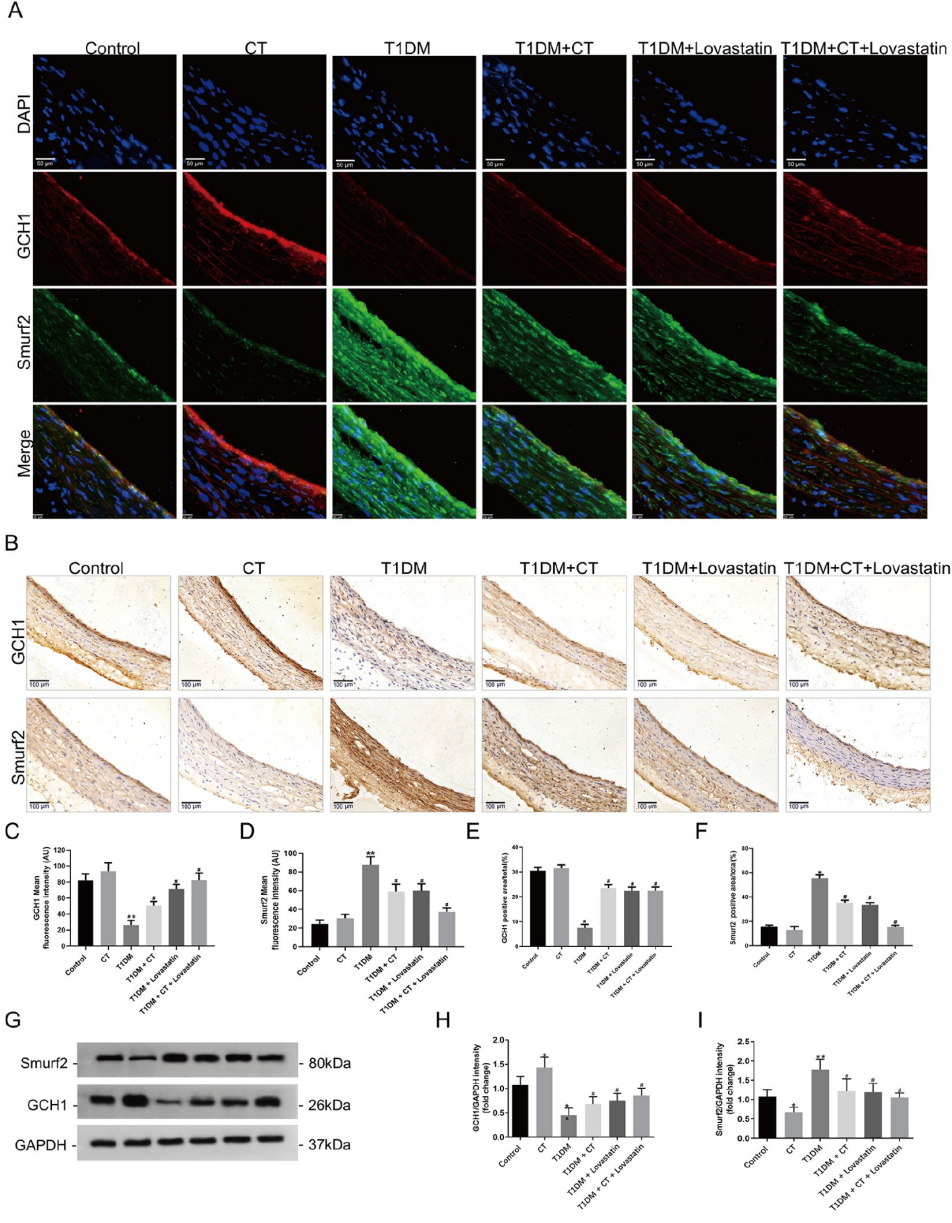
Figure 2 CT suppresses the protein expressions of Smurf2 and GCH1 in carotid arteries of T1DM rats (A) The immunofluorescence staining of Smurf2 and GCH1 in carotid arteries (Smurf2, green; GCH1, red) ( n=6). Scale bar: 50 μm. (B) The immunohistochemistry analysis of GCH1 and Smurf2 in carotid arteries ( n=6). Scale bar: 100 μm. (C,D) Quantitative analysis of immunofluorescence of GCH1 (Red) and Smurf2 (Green) in carotid arteries ( n=6). (E,F) Quantitative analysis of positive intensity of GCH1 and Smurf2 ( n=6). (G) The expression levels of Smurf2 and GCH1 proteins in carotid arteries were detected by western blot analysis ( n=6). (H,I) Quantitative analysis of (G). Data were analyzed by one-way ANOVA by Tukey post hoc tests or Bonferroni post hoc tests. All data are expressed as the mean±SEM. * P<0.05, ** P<0.01 vs Control group, # P<0.05 vs T1DM group.

GCH1, a pivotal enzyme catalyzing BH _4_ production, plays a crucial role in atherosclerotic endothelial dysfunction [7]. It has been reported that ubiquitination may be important for regulating GCH1 protein level and activity [ 42, 43] . In the search for the E3 ligase of GCH1, Smurf2 ranked first among several E3 ubiquitin ligases that displayed negative expression correlation with GCH1 in carotid arteries. In the T1DM-induced ED rat model, we examined the levels of Smurf2 and GCH1 proteins in vascular tissues by immunofluorescence staining and immunohistochemistry analysis. As shown in Figure 2A, the immunofluorescence staining results showed a decrease in the fluorescence intensity of Smurf2 protein and an increase in the fluorescence intensity of GCH1 protein in the CT-intervention group compared with the T1DM group. Additionally, the immunohistochemistry analysis and western blot analysis results verified the above results ( Figure 2B‒I, *P*<0.05). These findings suggest an inverse relationship between GCH1 and Smurf2 levels during CT-mediated intervention *in vivo*.

### CT significantly reduces apoptosis levels in *vivo* and in *vitro*

**Figure 3:**
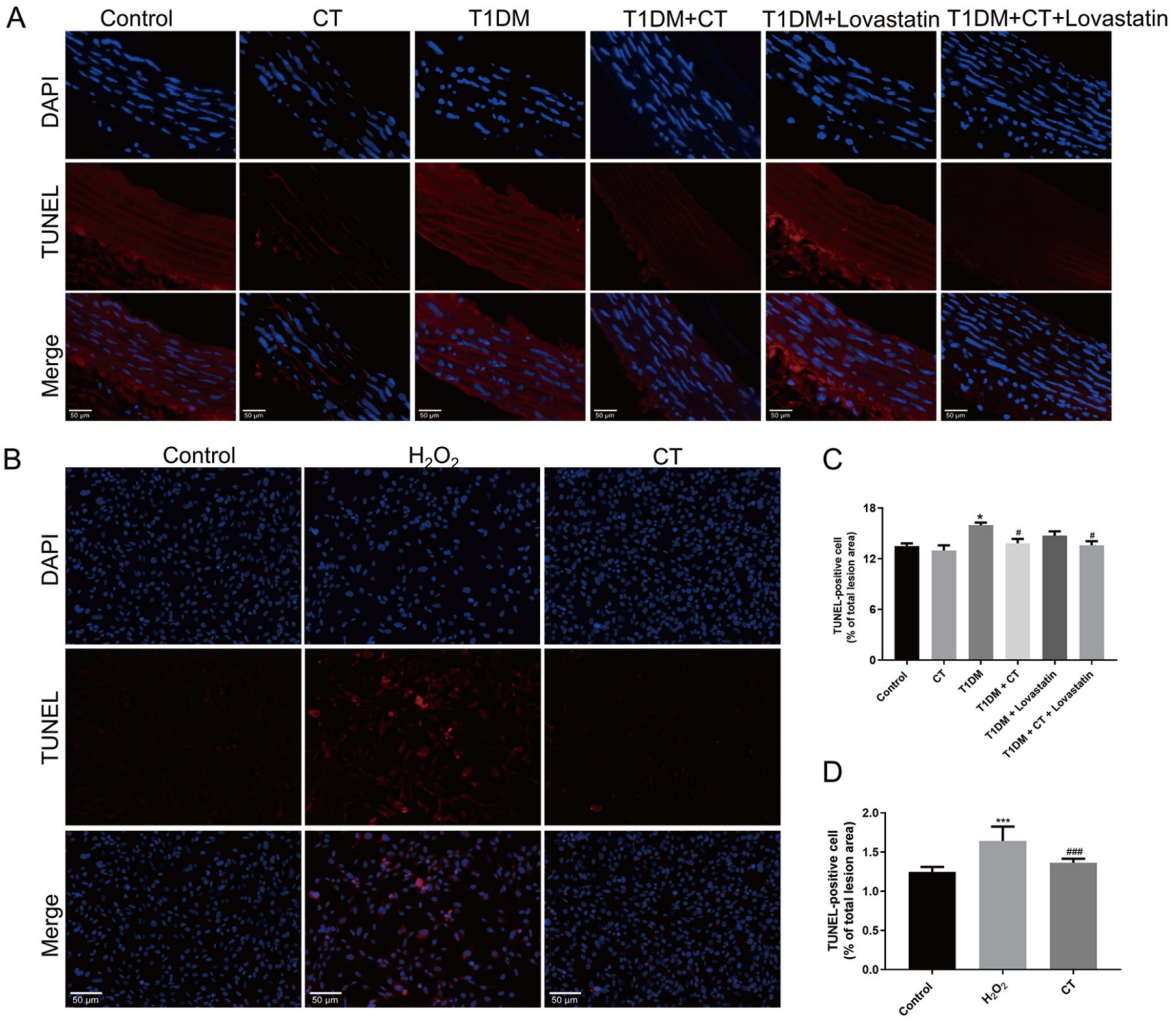
Figure 3 CT inhibits apoptosis of endothelial cells *in vivo* and *in vitro* (A,B) TUNEL staining in carotid arteries ( n=6) and HUVECs ( n=3). Scale bar: 50 μm. (C) Quantitative analysis of positive cell apoptosis rate in carotid arteries ( n=6). (D) Quantitative analysis of positive cell apoptosis rate in HUVECs ( n=3). Data were analyzed by one-way ANOVA by Tukey post hoc tests or Bonferroni post hoc tests. All data are expressed as the mean±SEM. * P<0.05, *** P<0.001 vs Control group, # P<0.05, ### P<0.001 vs T1DM group or H 2O 2 group.

In carotid arteries, lovastatin and/or CT treatment reduced apoptosis in T1DM rat vascular tissues compared to the T1DM group, as revealed by TUNEL staining ( Figure 3A,C, *P*<0.05). Similarly, in HUVECs exposed to H _2_O _2_, CT intervention significantly reduced the apoptosis rate compared to the H _2_O _2_ group ( Figure 3B,D, *P*<0.05). CT exhibited a mitigating effect on apoptosis *in vivo* and *in vitro*.

### Smurf2 co-immunoprecipitates and colocalizes with GCH1

**Figure 4:**
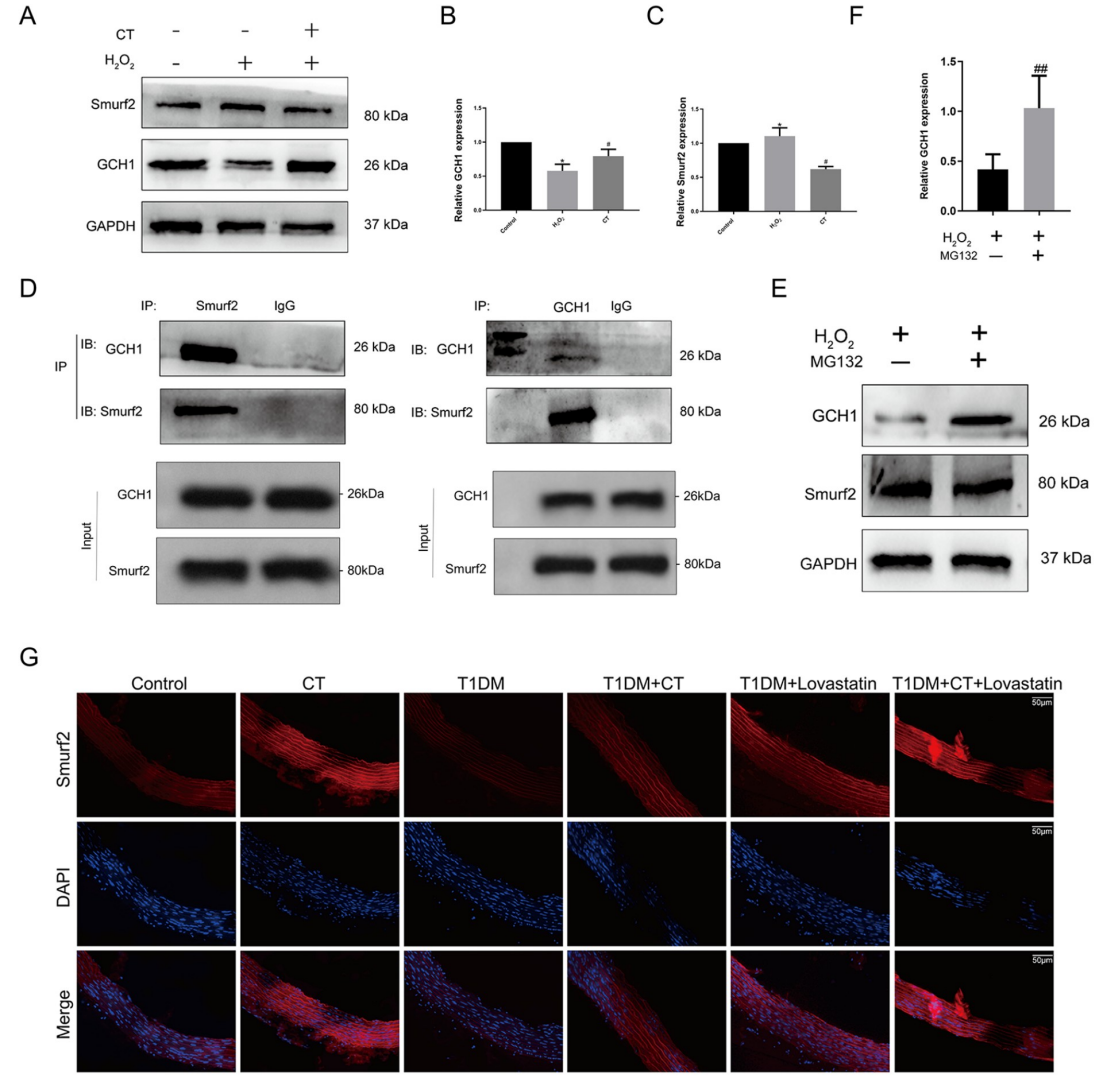
Figure 4 The interaction of Smurf2 with the GCH1 protein was determined by co-immunoprecipitation experiments (A) CT suppressed the protein expressions of Smurf2 and GCH1 in HUVECs ( n=3). (B,C) Quantitative analysis of (A) ( n=3). (D) The interaction of Smurf2 with GCH1 was determined by reciprocal coIP assay in HUVECs. Input groups were used as an internal protein loading control ( n=3). (E) Supplementation with the proteasome inhibitor MG132 can prevents Smurf2-induced degradation of GCH1 ( n=3). (F) Quantitative analysis of (E) ( n=3). (G) Immunofluorescence staining of eNOS in carotid arteries ( n=6). Scale bar: 50 μm. Data were analyzed by one-way ANOVA by Tukey post hoc tests or Bonferroni post hoc tests. All data are expressed as the mean±SEM. * P<0.05 vs Control group, # P<0.05, ## P<0.01 vs T1DM group or H 2O 2 group.

*In vitro* experiments confirmed that CT modulated GCH1 and Smurf2 protein expressions, consistent with the *in vivo* findings ( Figure 4A‒C). Co-immunoprecipitation assays demonstrated the endogenous interaction between GCH1 and Smurf2 proteins in HUVECs ( Figure 4D, *P*<0.05). Additionally, the proteasome inhibitor MG132 prevented Smurf2-mediated GCH1 degradation ( Figure 4E,F), indicating that Smurf2 promotes GCH1 degradation via the proteasome-mediated ubiquitination pathway.

### CT upregulates T1DM-induced eNOS in thoracic arteries

To verify that CT could negatively regulate the expression of Smurf2/GCH1 protein, we again used immunofluorescence staining and immunohistochemistry analysis to detect the expression of the GCH1 downstream protein eNOS. As shown in Figure 4G, the distribution and expression of eNOS in the aorta were detected by immunofluorescence staining in this experiment. The expression levels of eNOS were more prominently distributed in the lovastatin group, CT group, and the combined treatment group. However, the distribution of eNOS in the T1DM group decreased. These results indicate that CT improves vascular endothelial function induced by T1DM mellitus by enhancing the GCH1/eNOS signaling pathway.

## Discussion

In recent years, the escalating prevalence and impact of diabetic-associated cardiovascular diseases have imposed significant global societal and economic burdens [ 44– 46] . Endothelial cells, constituting the innermost layer of blood vessels, play a pivotal role in these complications. ED causes multi-organ impairment, which may lead to the death of patients with T1DM [ 47– 49] . Therefore, we mainly investigate the effect of CT on endothelial cells in *vitro* and in *vivo*. In this study, we observed that CT administration remarkably attenuated the apoptosis responses to H _2_O _2_. Subsequently, the GCH1/BH _4_/eNOS signaling pathway was extensively studied in T1DM-induced ED, mainly in endothelial cells.

Steady-state imbalance of NO and ROS may lead to endothelial-dependent impairment of vasodilation and enhancement of inflammatory responses, oxidative stress and HUVECs injury [ 20, 50] . eNOS is the crucial rate-limiting enzyme for NO synthesis, converting L-arginine into NO [51]. However, under pathological conditions, eNOS can undergo uncoupling, promoting the generation of superoxide instead of NO [ 19, 52] . eNOS uncoupling is partially promoted by GCH1 downregulation, which leads to dysfunction of BH _4_ secretion and impaired NO synthesis [53]. Enhanced GCH1-mediated eNOS recirculation alleviates T1DM-induced ED [54]. In addition, exogenous zinc supplementation restores diabetic endothelial dysfunction via upregulating GCH1 [5]. In the present study, T1DM-mediated induction of HUVECs decreased protein expression levels of GCH1 and eNOS, thus supporting the abnormal increase in ROS levels in HUVECs. CT significantly increased protein expression levels of GCH1 and eNOS and BH _4_ secretion in HUVECs, thus attenuating ROS generation and cell apoptosis, suggesting that CT improves oxidative stress, cell apoptosis and impairs BH _4_ secretion via the GCH1/eNOS signaling pathway.

Previously, we found that GCH1 played pivotal roles in regulating the increase in BH _4_ and NO levels in HUVECs [55]. This study identified Smurf2, a HECT type ubiquitin ligase, as an E3 ligase of GCH1, which reduces GCH1 protein level through proteasomal degradation. GCH1 mediates Smurf2-dependent BH _4_ in metabolism and NO production in HUVECs. Therefore, we revealed a novel metabolic role of Smurf2 by acting as an E3 ligase for GCH1 in HUVECs.

Smurf2 has been reported to play a dual role in cancers by functioning as both tumor promoter and suppressor by regulating the stability of proteins in tumorigenesis [ 22, 56– 60] . However, the role of Smurf2 in regulating endothelial damage in vascular endothelial cells is unclear. Smurf2 was originally identified as a regulator for the TGF-β signaling pathway [22]. TGF-β ws reported to regulate the expression of Smurf2 [ 61, 62] . Here we found that CT reduced Smurf2 protein level and increased GCH1 protein level in HUVECs. CT has been reported to ED induced by atherosclerosis [63]. However, it remains unclear whether CT regulates GCH1 level or activity in HUVECs. Our findings suggest a possible mechanistic link between Smurf2 and GCH1 by showing that CT may promote GCH1 stabilization through decreasing Smurf2 levels. The upstream signal regulating the activity of the CT-Smurf2-GCH1 axis requires further study.
